# LAMN as a differential diagnosis for abdominal pain: a case report from Syria

**DOI:** 10.1093/jscr/rjab585

**Published:** 2022-01-13

**Authors:** Joudi Tarabishi, Alma Douedari, Tahreer Almasalmeh, Mario Tarzi

**Affiliations:** Faculty of Medicine, University of Aleppo, Aleppo, Syria; Faculty of Medicine, University of Aleppo, Aleppo, Syria; Department of Surgery, University of Aleppo, Aleppo, Syria; Faculty of Medicine, University of Aleppo, Aleppo, Syria

## Abstract

Low-grade appendiceal mucinous neoplasms (LAMNs) are papillary or flat mucinous tumors with low-grade cytologic atypia found in <0.3% of appendectomy specimens among older population. They are the most frequent source of pseudomyxoma peritonei. They can be easily misdiagnosed, due to unspecific symptoms, with acute appendicitis, retroperitoneal tumors or adnexal mass. Macroscopically, the appendix may appear normal or be variably dilated. Microscopic study determines whether the studied specimen is LAMN or mucinous adenocarcinomas. We report a 77-year-old patient presented with 15-day abdominal pain accompanied with chills and hyperthermia. Decision was made for right hemicolectomy as a result of the findings on ultrasound and computed tomography scan. Diagnosis was made after the pathologic study, which revealedLAMN.

## INTRODUCTION

One of the most common types of specimens received in laboratory is vermiform appendices. In juvenile population, most specimens show standard features of acute pyogenic and necrotizing appendicitis, whereas in older population, low-grade appendiceal mucinous neoplasms (LAMNs) should be considered as a differential diagnosis, even they are found in <0.3% of appendectomy specimens, since they can be easily misdiagnosed due to unspecific symptoms like abdominal pain, nausea, vomiting and palpable mass in the right iliac fossa [1–4]. LAMNs account nearly 1% of gastrointestinal neoplasms and are characterized by villous or flat proliferation of mucinous epithelium with low-grade cytologic atypia and absence of destructive invasion leading to diverticulum-like structures [1, 4–6].

These tumors are not uncommon and are the most frequent source of pseudomyxoma peritonei (PMP), a unique peritoneal malignancy characterized by slow growth of tumor in the peritoneal cavity [7, 8].

## CASE PRESENTATION

A 77-year-old patient presented with a 15-day abdominal pain unresponsive to analgesics. He complained of chills and hyperthermia with one peak at night, while he did not mention any change in bowel habits. The patient had no medical history.

Clinical examination revealed a tender abdomen and pain located within the right iliac region. No abdominal masses were tangible.

Abdominal auscultation indicated a normal bowel motility. Digital rectal examination ‘DRE’ showed stool remaining on the probing finger. The patient had low blood pressure ‘110/70’ with a pulse of 80 beats\min. A number of laboratory tests were made (Table 1).

**Table 1:** Laboratory results

Laboratory results		
Test	Result	Normal range
Hemoglobin	12.9 g/dl	13.5–17.5 g/dl
White blood cell	91 000 m/mm^3^	4500–11 000 m/mm^3^
Neutrophils	71.6%	40–60%
Platelets	276 000 10*9/l	150 000–450 000 10*9/l
C-reactive protein	45 mg/l	<3 mg/l
Glucose	140 mg/dl	70–110 mg/dl
Urea	27 mg/dl	7–20 mg/dl
Creatinine	0.86 mg/dl	0.6–1.2 mg/dl
Amylase	27 U/l	23–85 U/l
Prothrombin time	14.2 seconds	11–12.5 seconds
Activated clotting time	90% seconds	70–120 seconds
International normalized ratio	1.12	0.8–1.1
Sodium	134 mEq/l	135–150 mEq/l
Potassium	4.8 mEq/l	3.5–5 mEq/l
Albumin	3.5 g/l	3.4–5.4 g/l
Weinberg	1/40	

Patient has A^+^ bloodtype.

These results are indicative of an inflamation of the retroperitoneal tumor that is depicted on the ct scan.

Echography depicted a dense fluid-filled mass of 10 cm diameter in the right iliac, the wall was not perfused.

**Figure 1:**
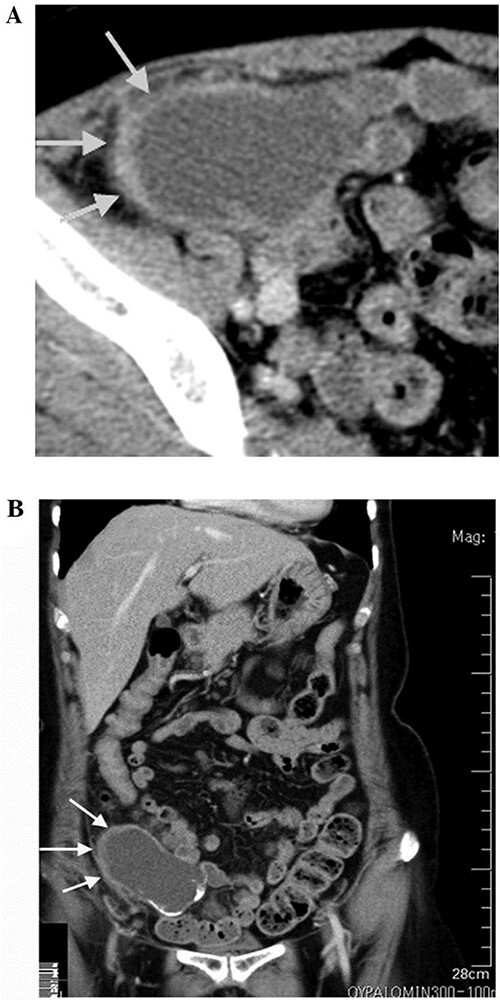
CT scan showing lobulated cystic formation in the right iliac vessels level with an extension to the upper margin of the pelvis. This finding is suspicious, so a decison was made for excision of the tumor and to be sent to pathology department to confirm the diagnosis.

Computed tomography (CT) scan was performed after perfusion of a radiopaque substance through the vein in both arterial and venous phases. It exhibited a 14-cm lobulated cystic formation in the level of the right iliac vessels with an extension to the upper margin of the pelvis. The wall of the cyst was thick and calcinated (Fig. 1).

Both echography and CT scan displayed normal findings for other inner organs within the abdominal cavity and confirmed the absence of free fluid.

**Figure 2:**
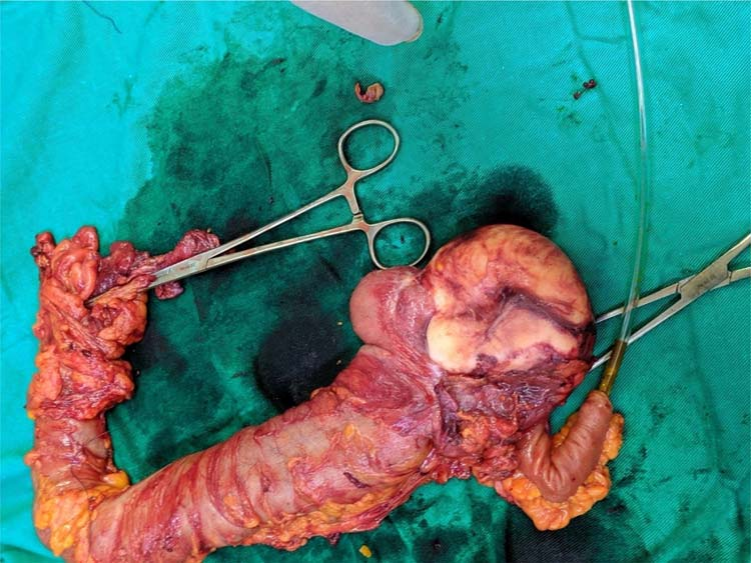
A gross appearance of the right excised colon.

An agreement of the surgical board to perform a right hemicolectomy (Fig. 2). The pathologic study made the diagnosis of a LAMN (Misdraji Classification), previously known as mucinous cystadenoma with low-grade dysplasia. It also stated normal findings regarding other components of the specimen.

## DISCUSSION

LAMNs represent a relatively homogeneous group of papillary or flat mucinous tumors with low-grade cytologic atypia analogous to low-grade dysplasia in other parts of the gastrointestinal tract [5]. LAMNs are classified as low-grade adenocarcinomas, since they possess the potential for peritoneal spread and thus may cause death [6]. Recently, experts consensus that LAMNs should be classified based on the tumor nodes and metastasis criteria. Presently, LAMNs are staged using the same system as other appendiceal adenocarcinomas according to the seventh edition of the American Joint Committee on Cancer Staging Manual. The staging is depending on the depth of invasion through the appendiceal wall. Nevertheless, assessing depth of invasion in LAMN is challenging, primarily owing to their lack of destructive invasion and the propensity for mucin extravasation. Furthermore, the currently used T categories may not provide prognostic value in LAMN. Thus, a staging system specifically for LAMN that conveys prognostic information is necessary for guiding clinical management [6].

Macroscopically, the appendix may appear normal or variably dilated. Dilated LAMNs typically show thin fibrous walls with calcification of the wall and/or the intraluminal mucin. Most patients with PMP present with grossly apparent rupture with extrusion of mucus. Severe inflammatory response to mucus might disguise the presence of mucin around the appendix, stimulating ruptured appendicitis [7].

Microscopically, LAMNs were classified when demonstrating low-grade cytologic atypia and minimal architectural complexity, but when demonstrating whether destructive invasion of the appendiceal wall, high-grade cytologic atypia or complex epithelial proliferation, they were classified as mucinous adenocarcinomas [5]. Histological evidence of LAMN includes atypical glandular cells and epithelial cells with ‘pushing invasion’ of malignant cells creeping into the appendiceal wall with possible diverticular formation [4] leading to easily misdiagnosing with appendiceal diverticulosis disease.

LAMNs are characterized by frequent KRAS mutations and do not generally show microsatellite instability or BRAF mutation. They also commonly contain GNAS mutations, which are unusual in colorectal neoplasms [7].

LAMNs are associated with diverticula, herniations, dissections, rupture and ovarian mucinous tumors. The most significant complication is seeding of mucin into the adjacent peritoneum, causing PMP [4].

Often, this malignancy is misdiagnosed as acute appendicitis, retroperitoneal tumors in the right iliac fossa or an adnexal mass. Imaging tools used for diagnosis are ultrasound and CT. CT findings include cystic dilation within the appendiceal lumen with wall calcifications and irregular appendiceal wall thickening as demonstrated in our case. Grossly, specimens include hyalinization and fibrosis of the appendiceal wall with a grossly swollen appendix secondary to mucinous accumulation [4].

The optimal approach remains controversial, preferably the surgical approach, adjuvant therapy, follow-up duration and imaging technique. The goal of management of LAMN includes the prevention of rupture, seeding and development of PMP as performed in our case [4].

Overall, further studies are needed for a more definitive method of diagnosis, treatment and monitoring of LAMN. This case presents the importance of developing a high index of suspicion regarding the development of appendiceal malignancies and choosing the appropriate surgical or medical treatment modality to prevent recurrence, seeding, and development of PMP [4].

## AUTHORS’ CONTRIBUTION

Conception and design: J.T., A.D. and M.T. Analysis and interpretation of the data: J.T., M.T. and A.D. Drafting of the article: J.T. and A.D. Critical revision of the article for important intellectual content: M.T. and T.A. All authors read and approved the final version of the manuscript.

## CONSENT FOR PUBLICATION

An informed consent was signed by the patient.

## CONFLICT OF INTEREST STATEMENT

We have no conflict of interest.

## FUNDING

There are no funding sources.
